# Identification of Antitumor Constituents in Toad Venom by Spectrum-Effect Relationship Analysis and Investigation on Its Pharmacologic Mechanism

**DOI:** 10.3390/molecules25184269

**Published:** 2020-09-18

**Authors:** Ji-Heng Wu, Yue-Ting Cao, Hong-Ye Pan, Long-Hu Wang

**Affiliations:** College of Pharmaceutical Sciences, Zhejiang University, Hangzhou 310058, China; wujiheng@zju.edu.cn (J.-H.W.); caoyueting201314@zju.edu.cn (Y.-T.C.); 11819004@zju.edu.cn (H.-Y.P.)

**Keywords:** toad venom, spectrum-effect relationship, chemometrics, apoptosis

## Abstract

(1) Background: Toad venom (Bufonis Venenum, known as ‘Chansu’ in Chinese), the secretion of the ear-side gland and skin gland of Bufo gargarizans cantor or Duttaphrynus melanostictus Schneider, has been utilized to treat several diseases in China for thousands of years. However, due to the chemical variability of the components, systematic chemical composition and the key pharmacophores in toad venom have not yet fully understood. Besides, it contains a variety of effective compounds with different physiological activity and chemotypes, mainly including alkaloids, bufogenins, bufotoxins, and so on. The recent pharmacological researches have demonstrated that several bufogenins have remarkable pharmacological effects, such as anti-inflammatory, analgesic effects, and anti-tumor effects. Aim of the study: To identify the bioactive compounds and pharmacophores originating from toad venom based on analyzing spectrum-effect relationship by chemometrics and to explore the anti-cancer mechanism primarily. (2) Materials and methods: Fingerprint of the 21 batches of samples was established using HPLC (High Performance Liquid Chromatography). The anti-tumor activity of extracts were determined by in-vitro assays. Chemometric analysis was used to establish the spectrum-effect model and screen for active ingredients. Pharmacodynamic tests for the screened active compound monomers were conducted with in-vitro assays. Further anti-tumor mechanisms were investigated using western blot and flow cytometry. (3) Results: The established spectrum-effect model has satisfactory fitting effect and predicting accuracy. The inhibitory effect of major screened compounds on lung carcinoma cells A549 were validated in vitro, demonstrating that arenobufagin, telocinobufogenin, and cinobufotalin had significant anti-tumor effects. Through further investigation of the mechanism by western blotting and flow cytometry, we elucidated that arenobufagin induces apoptosis in A549 cells with the enhanced expression of cleaved PARP (poly (ADP-ribose) polymerase). These results may provide valuable information for further structural modification of bufadienolides to treat lung cancer and a method for discovery of anti-tumor active compounds. Conclusions: Our research offers a more scientific method for screening the principal ingredients dominating the pharmacodynamic function. These screened compounds (arenobufagin, etc.) were proven to induce apoptosis by overactivation of the PARP-pathway, which may be utilized to make BRCA (breast cancer susceptibility gene) mutant cancer cells more vulnerable to DNA damaging agents and kill them.

## 1. Introduction

Lung cancer is one of the most common cancers in humans with high incidence and mortality rate. Non-Small Cell Lung Carcinoma (NSCLC), the most common form of lung cancer, comprises of approximately 85% of all cases of lung cancer [1,2]. Natural medicines have shown attractive potential for preventing and treating diseases for centuries and have contributed to the development of modern medicine [3]. Toad venom, the dried white secretion from the postauricular and skin glands of Bufo gargarizans cantor, is well known for treating many kinds of cancer [4]. According to traditional records, the main efficacy of toad venom is in detoxification, as an analgesic, etc. [5]. The medicine currently used in clinical practice in China are mainly complexes that have originated from toad venom [6,7,8]. Modern medicines composed of toad venom include Huachansu injection, and Shexiang Baoxin Pills, among others. Huachansu injection, prepared from a water extract of dried toad venom, has long been used to treat various cancers of the digestive system [9,10]. It has also shown a reversal effect on multi-drug resistance (MDR) of acute myeloid leukemia cells [11]. In combination with chemotherapy, Huachansu injection enhances curative effects and diminishes the side-effects of chemotherapy [12]. Shexiang Baoxin Pills, which are composed of Moschus, Bufonis Venenum, etc., have been commonly used for cardiovascular diseases, like unstable angina pectoris [13,14]. Because of the complexity of toad venom, both of these medicines have shown drug-related adverse effects, including cardiac toxicity, hematologic toxicities, mucocutaneous toxicities, and gastrointestinal toxicities, thus limiting their use [15]. There is, therefore, a great need to distinguish the active ingredients in toad venom and further study its underlying molecular mechanism. 

Bufogenin and bufotoxin, as major components of toad venom, are considered to be the main bioactive constituents, which exert various pharmacological effects with different mechanisms. Ma [16] reported that arenobufagin has anticancer influences on several non-small-cell lung cancer (NSCLC) cells through activation of Noxa (the pro-apoptosis protein)-related signaling pathways and promotes apoptotic cell death in human NSCLC cells. Kai [17] held that cinobufotalin showed obvious inhibitory effects against lung cancer cells without inducing significant cell apoptosis, and Zhang [18] discovered that arenobufagin induced apoptosis and autophagy in human hepatoma carcinoma cells through PI3K/AKt/mTOR pathway inhibition. As shown by these studies, different bufadienolides conducted antitumor functions by regulating different cell signaling pathways. As a result, different bufadienolides have distinct inhibitory effects on diverse kinds of cancer, which awaits further exploration and utilization. It is thus urgent to identify the active ingredients in toad venom.

Spectrum-effect relationship analysis is an effective method to clarify active components in complex mixtures. By combining the characteristic fingerprint and pharmacodynamics information processed by chemometric methods, effective components can be screened.

Chromatographic fingerprint is an effective method to evaluate the consistency and quality of traditional Chinese medicines (TCMs), which could reveal the chemical characteristics of samples to a certain extent [19]. Multiple techniques including HPLC, gas chromatography (GC), etc. have been used to construct specific fingerprints for recognition of complex compounds of TCMs. Among them, HPLC is a broadly applied method owing to its high sensitivity and accessibility [20]. HPLC-MS is an analytical technique mainly used for identification of chemical structures.

While fingerprint analysis is a useful method for chemical analysis of complex matrices [21], it does not involve the identification of components that play leading roles in pharmacology activity. In this paper, with the aim to research the correlation between the biological activity of toad venom and the fingerprint, multivariate chemometrics techniques (including orthogonal partial least squares (OPLS), canonical correlation analysis (CCA), and gray relationship analysis (GRA)) were employed.

PCA is a method to analyze and simplify data set by reducing its dimensionality, and keeping the largest contribution to the variance of the data set [22,23]. OPLS is a generic method to build a model of the observed data in order to analyze the relationship between two groups of variables and selecting the key variables [24]. Processed by OPLS, variable importance in projection (VIP) reflects the loading weights of each independent variable to the dependent variable. When VIP > 1, the independent variable is a significant factor in interpreting the dependent variable [24]. GRA, which originated from the grey system theory proposed by Deng [25] in the 1980s, is suitable for manipulating complex interrelationships between multiple factors and variables. GRA results can be provided as a ranking sequence that reflects the order of correlation among dependent and independent factors [26]. Correlation analysis is a statistic analytical method to research the linear relation between variables; the correlation coefficients are used to measure the degree of correlation of the variables [19].

These analysis methods were used to establish the spectrum-effect relationship between the peak area of the fingerprint and the results of anti-cancer activity study, respectively. We isolated seven bufadienolides (hellebrigenol, arenobufagin, hellebrigenin, 19-oxo-cinobufotalin, telocinobufogenin, 19-oxo-cinobufagin, cinobufotalin) and evaluated the inhibitory effect of the compounds with relatively high content on two non-small cell lung cancer cells in vitro. Herein, we found out the key pharmacophores of bufadienolides and further investigated the possible mechanism of arenobufagin, which is most significant correlated with antitumor activity.

## 2. Results and Discussion

### 2.1. HPLC Fingerprints and Similarities Analysis

#### 2.1.1. Establishment of the Fingerprint of Toad Venom

**Figure 1:**
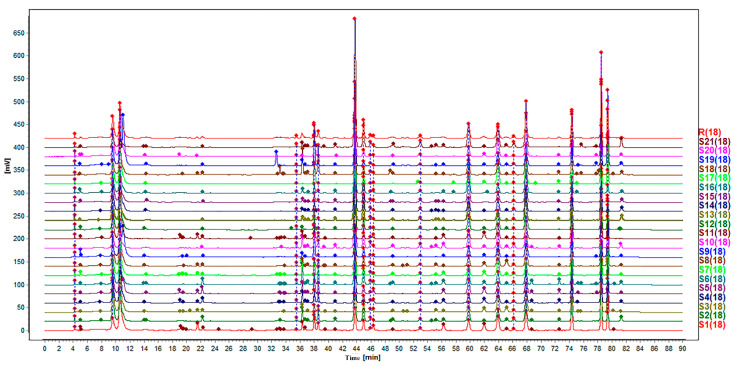
HPLC fingerprints of the 21 batches of toad venom extracts.

**Figure 2:**
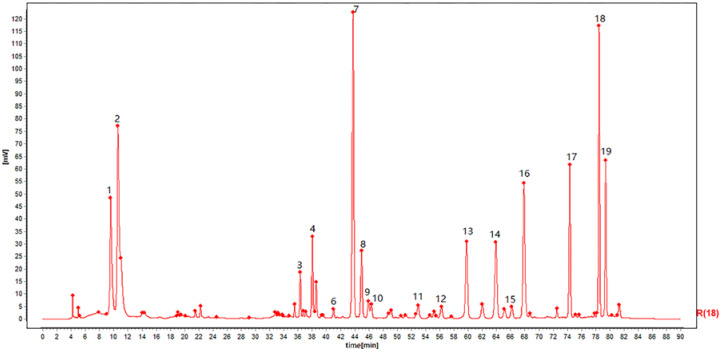
The reference atlas of the toad venom extracts.

The HPLC fingerprints for 21 batches of toad venom samples are shown in Figure 1, which was matched by the Similarity Evaluation System for Chromatographic Fingerprints (version 2012.130723). Peaks with good separation and relatively large areas were determined as common peaks. Therefore, 19 peaks were matched by comparing their peak shape and HPLC retention time, which account for more than 90% of the total chromatographic peak area (Figure 2). The areas of 21 batches of toad venom extracts samples are listed in Table 1. The peak area for peaks lacking in chromatograms was defined as “0”. The RSDs of the RPAs and RRTs were determined for the 19 characteristic chromatographic peaks within a run time of 90 min. As seen from the table, the same ingredients from different batches of samples have different contents, showing quality differences between the extracts.

#### 2.1.2. Similarity Analysis of the HPLC Fingerprints

To verify quality differences between the samples, similarities between the entire chromatographic profile of the 21 batches of toad venom and the reference chromatogram were analyzed by the Similarity Evaluation System for Chromatographic Fingerprints (version 2012.130723). The similarity range between each batch of toad venom and the standard was 0.771–0.984, indicating certain differences among the 21 batches (Table 2).

#### 2.1.3. PCA Results

In view of the complex composition of toad venom, we used principal component analysis, a dimension reduction method, to transform complex multivariables to a few comprehensive indices. The factor extract and factor rotation for each variable are shown in Table 3 The eigenvalue of the first six principal components in the PCA of the toad venom extracts was large (average > 1). According to the principal determining number of the components, the contribution rate of more than 85% was set as the principal component extract standard; the first six principal components were extracted for analysis. The first five components account for 89.575% information of the overall index (Table 4).

The aboriginal data represented by six principal components were Y1, Y2, Y3, Y4, and Y5. The quality appraise model of toad venom was established as the comprehensive evaluation function of toad venom:

Y = (Y1 × 40.507 + Y2 × 18.942 + Y3 × 14.137 + Y4 × 8.943 + Y5 × 7.047)/89.575(1)

The quality of toad venom from different batches was assessed by calculating the comprehensive scores using the expression above. The higher the comprehensive score, the better the quality of the products is.

### 2.2. Anti-Tumor Activity

**Figure 3:**
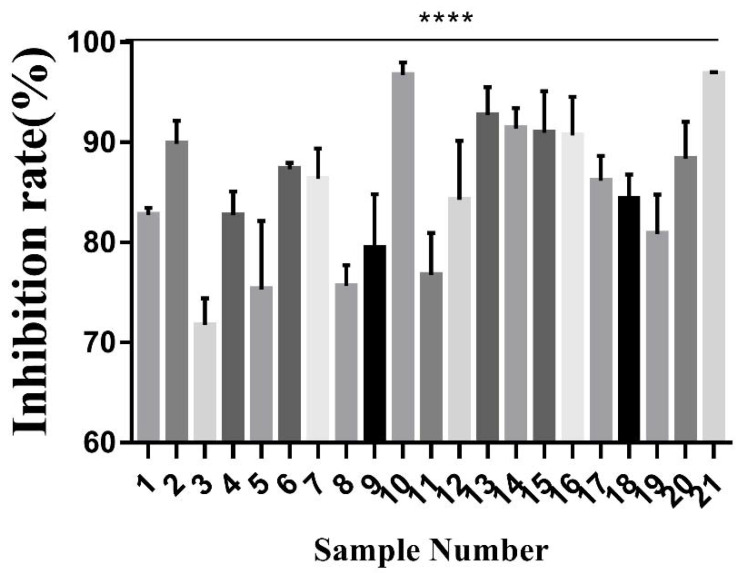
In vitro anticancer activities of 21 batches of toad venom extracts (A549 cells were incubated with 100 ng/mL toad venom extracts for 72 h, and cell viability was examined by MTT assay). Ordinary one-way ANOVA, **** *p* < 0.00001 indicates a significant difference versus the control group.

Cell proliferation assay was applied to distinguish the pharmacodynamics and in vitro cytotoxicity of the toad venom extracts on A549 cells. As shown in Figure 3 and Table 5, the lowest ratio was 71.752% (sample 3), and the highest ratio was 96.811% (sample 21). The test results showed a significant difference in the pharmacodynamics of these extracts. These data provide a basis to study the screen of the main active ingredients. 

### 2.3. Identification of Active Constituents

#### 2.3.1. GRA (Grey Relation Analysis) Results

To identify the active components, firstly, we used the pharmacodynamic indexes as the reference series, and the 19 common peaks as the compared series. After the normalization of the original data by “Z-SCORE”, the gray relational coefficients for each common peak were obtained. As shown in Table 6, the contribution of the components of toad venom on the pharmacodynamics are sorted from high to low: 7 > 8 > 6 > 11 > 15 > 9 > 13 > 1 > 16 > 14 > 4 > 12 > 5 > 3 > 17 > 18 > 10 > 2 > 19. The relational grade of components **7** and **8** was greater than 0.8, which means that they had a significant correlation with the pharmacodynamic activity. Components **6**, **11**, **15**, **9**, **13**, **1**, and **16** had correlation coefficients between 0.7 and 0.8, which means a close correlations to cancer cell proliferation activity. The correlation degree of the remaining components, except for component **19**, was between 0.6 and 0.7. It can be derived from Table 1 and Table 2 that the samples with relatively higher areas of peaks of 7, 8, 6, 11, 15, 9, 13, 1 and 16 show better pharmacodynamic activity. These results agree that these ingredients contribute to pharmacological effects with different degrees.

#### 2.3.2. CCA (Canonical Correlation Analysis)

Secondly, CCA was applied to assess the relationship between the areas of 19 peaks in fingerprints and the proliferation inhibition rate. The Pearson correlation of the two groups of variables was calculated using canonical correlation analysis by SPSS software. The results are shown in Table 7. A positive correlation coefficient suggests a positive correlation with the antitumor activity, while a negative correlation coefficient indicate that it is negatively correlated with the antitumor activity. As a result, compounds **6**, **7**, **8**, **11**, **13**, **15**, **16**, and **14** had a strong correlation with the inhibition rate. In conclusion, these components might be the main components inhibiting the proliferation of A549. Here, we noticed that the coefficient of some components were negative, such as 19 peak, which was resibufogenin. However, according to known references [27], resibufogenin also shows some antitumor effects on A549 cells with IC50 of about 25 nM [28], which means that resibufogenin also has some degree of antitumor activity. To explain this result, we analyzed the relationship between the peak areas of 6, 7, 8, 11, 13, 15, and 16, the most significant peaks according to our analysis, and peak 19 with CCA. The results showed that the coefficients between them are −0.709; −0.704; −0.742; −0.466; −0.692; −0.447; −0.471, respectively, which means that they had a significant negative correlation. According to the presented reference [16], the IC50 value of arenobufagin in A549 is less than 10 nM, which indicates a much higher antitumor activity than resibufogenin. Thus, when resibufogenin content is higher, the contents of the seven components mentioned above are lower, which lead to a reduction in antitumor effects and a negative coefficient between them.

#### 2.3.3. OPLS (Orthogonal Partial Least Squares) Analysis

**Figure 4:**
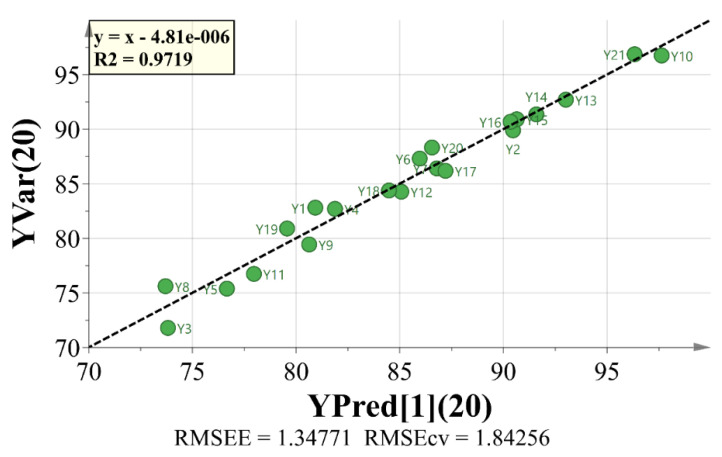
Graphical representation of OPLSR model—calibration model.

**Figure 5:**
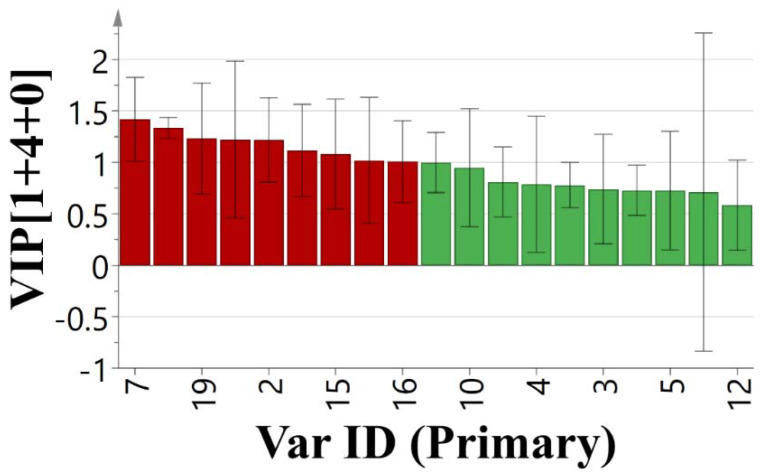
Graphical representation of OPLSR model—VIP plot.

Thirdly, the spectrum-effect relationship between the peak area of the fingerprint and the inhibition rate of A549 cells was analyzed by OPLS. The data of the peak area and the pharmacological test were transferred into the Microsoft program Simca-p 14.1 (Demo version). All variables were preprocessed by unit variance scaling before analysis. The calibration model presented in Figure 4 showed a good concordance between the predicted values and the actual values. The OPLS model with five principal components exhibited satisfactory fitting capacity (R_2_ = 0.972) and predictive ability (Q_2_ = 0.927), with a root mean square error of estimation (RMSEE) of 1.35 and a root mean square error from cross-validation (RMSECV) of 1.84. Using the variable importance in projection (VIP) plot, variables with a greater VIP score (larger than 1) are selected as the main active components with significant influence on anti-tumor activity. As presented in Figure 5, the main chemical components with greater VIP scores (marked by red bars), in descending order, were peaks 7, 8, 19, 11, 2, 6, 15, 13, and 16.

The characteristic components integrated by the above chemometric analysis were generally consistent. The intersections of OPLS, the correlation analysis, and gray correlation analysis results were components of 7, 8, 6, 11, 15, 13, and 16, which should be the main active components in toad venom that inhibit the proliferation of A549 cells.

### 2.4. Structural Identification by HPLC-TOF-MS

**Figure 6:**
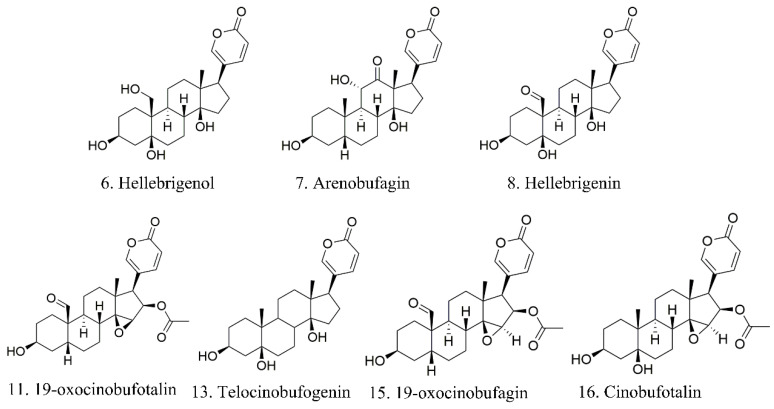
The structure of compounds identified in toad venom extract.

HPLC-ESI-Q-TOF-MS/MS with positive ion mode of ESI was used to qualitatively assign the structures of the above compounds. The MS data of seven identified active compounds are shown in Table 8, The fragmentation pathways and the typical MS/MS spectrums of the screened compounds are shown in the Supplementary Materials. The structural identification of the correlated peaks showed that 6 was hellebrigenol, 7 was arenobufagin, 8 was hellebrigenin, 11 was 19-oxo-cinobufotalin, 13 was telocinobufogenin, 15 was 19-oxo-cinobufagin, and 16 was cinobufotalin, respectively. The structures of the seven components are shown in Figure 6. Previous reports have shown that the basic bufadienolide skeleton of a steroidal A/B cis and C/D cis structure with a α- pyrone ring at position C17 is crucial to maintain the activity; the 5 β- hydroxy substituent increased the activity. According to the results, all of the characteristic peaks have minor groups at the C-1 position, the minor electron-donating group, hydrogen acceptor or donor substituent at the C-3site, which would exhibit a higher antitumor activity. Compound **7** with 11α-hydroxyl and 12-carbonyl groups exhibited the strongest inhibitory effects, illustrating that such structural character would contribute to cytotoxic activity, which is consistent with a related report [3]. The hydroxymethyl (electron-donating) at the C-10 position of compound 6 and aldehyde (hydrogen donor) at the C-10 position of compounds **8**, **11**, and **15** as the electron-donating group would enhance antitumor activity [3].

### 2.5. Confirmation of Effectiveness and Mechanism Research

**Figure 7:**
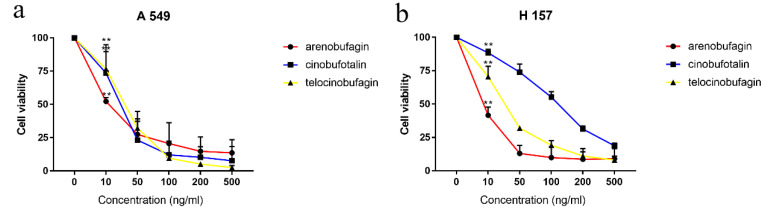
The inhibitory effects of arenobufagin, telocinobufagin, and cinobufotalin on A549 cells (**a**) and H157 cells (**b**) analyzed by 3-(4, 5-dimethylthiazol-2-yl)-2, 5-diphenyltetrazolium bromide (MTT) assay. ** Indicates statistical significance (*p* < 0.01).

**Figure 8:**
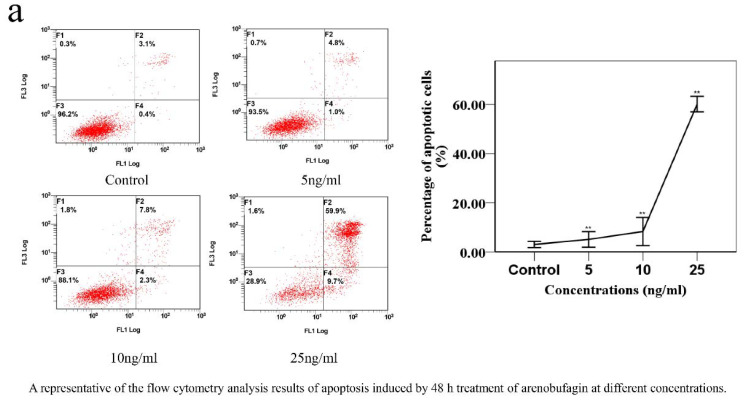

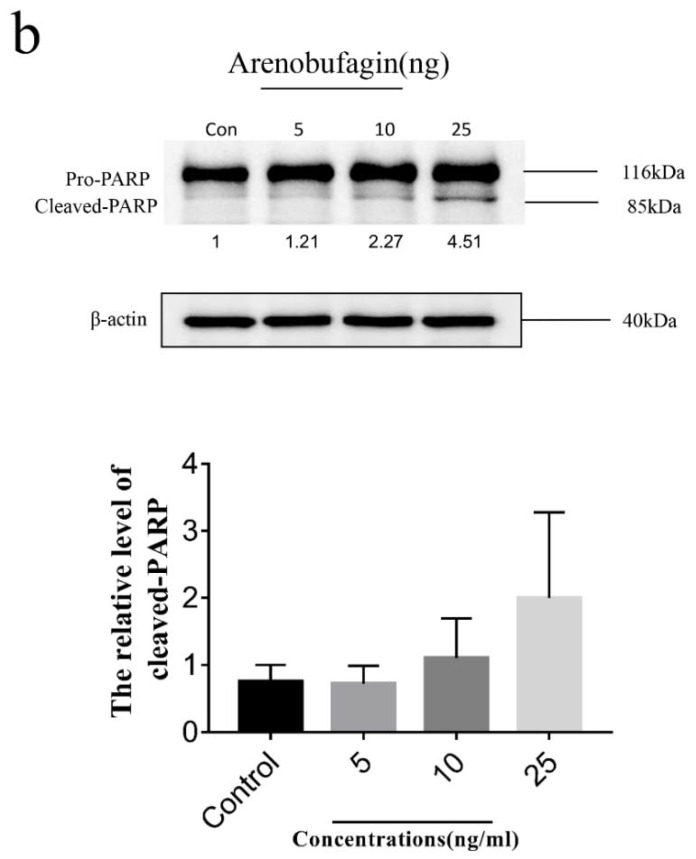
Arenobufagin induces apoptosis in A549 cells. A549 cells were incubated with arenobufagin for 48 h. (**a**) The effects of arenobufagin on apoptosis were analyzed by flow cytometry. (**b**) Detecting the protein expression levels of PARP by Western blotting.

To further verify the analysis results, the inhibitory effects of the screened compounds on the viability of the NSCLC cell lines (A549, H157) were detected by MTT assay. The results were represented as IC50 values (shown in Table 9 and Figure 7). All the bufadienolides tested showed a significant inhibiting effect on the proliferation of A549, H157 in a dose-dependent manner, illustrating that these compounds have an excellent anti-NSCLC effect. To explore the underlying mechanism, arenobufagin was selected to induce apoptosis in A549 cells. By staining cells with fluorescein annexin V-FITC and PI, it was further proved that arenobufagin treatment increased the frequency of apoptotic (annexin positive) cells in a dose-dependent manner (Figure 8a). Western blot analysis showed that the expression of the cleaved PARP was significantly enhanced after arenobufagin treatment. PARP is a group of nuclear enzymes that catalyze the transfer of ADP-ribose to target proteins [29]. It plays a significant role in many cellular processes, including regulation of chromatin structure, transcription, replication, recombination, and DNA repair [29]. The cleavage of PARP has been used as a marker of apoptosis in western blot analysis; our experimental results showed that arenobufagin induced the cleavage of PARP in a concentration-dependent manner (Figure 8b). In general, the cytotoxicity of bufadienolides in NSCLC may be associated with apoptosis, which means that arenobufagin has the potential to become a candidate for PARP inhibition.

## 3. Materials and Methods

### 3.1. Materials and Reagents

Twenty-one batches of toad venom derived from Bufo gargarizans Cantor were collected from Jiangsu China. The reference substances, including arenobufagin, telocinobufogenin, and cinobufotalin were provided by Yuanye Bio-Technology Co, Ltd. (Shanghai, China). The purity of all the substances was detected to be higher than 98% by HPLC-DAD. HPLC grade acetonitrile, formid acid, and ammonium formate were obtained from Aladdin Bio-Chem Technology Co, Ltd. (Shanghai, China). Ultra water was prepared by using a Milli-Q plus system (Millipore, Billerica, MA, USA).

### 3.2. Cell Culture

Human No-Small Cell Lung Cancer cell lines were obtained from Cobioer Biosciences (Nanjing, China). Cell lines A549 and H157 was cultured in RPMI-1640 supplemented with 10% foetal bovine serum in an incubator with 5% CO2 at 37°C. Cell lines were authenticated by short-tandem repeat genotyping performed by the Shanghai Bio Wing Applied Biotechnology Company (Shanghai, China), displaying identical morphology as cells provided by ATCC.

### 3.3. Preparation of Extracts and Standard Solutions

Each batch of toad venom was ground to 40 mesh, and precisely measured powder (5 g) was immersed in methanol (50 mL) and then extracted thrice by reflux for 1 h. The extract was filtered, combined, and concentrated in a rotary evaporator by evaporation and vacuum (60 °C water bath) and then dried in a vacuum freeze-drying machine. For the HPLC analysis, the precisely measured final filtrate was diluted with methanol to 25 mL, and then the mixture was filtered through a 0.22 μm filter before HPLC analysis. The final concentration was 2 mg/mL.

### 3.4. Instrumentation and Analysis Conditions

#### 3.4.1. HPLC Conditions

In our previous works, the HPLC analysis method with satisfactory selectivity and efficiency was established [30]. The HPLC analysis was performed with the Agilent 1200 system (Agilent Technologies, Tokyo, Japan) with an X Bridge reverse phase C18 column (4.6 × 250 mm, 5μm). The mobile phase was composed of 0.3% Acetic Acid-10mmol ammonium acetate water (A) and acetonitrile (B). The gradient program was 97–95% A for 0-7 min, 95–95% A for 7–11 min, 95–85% A for 11–13, 85–85% A for 13–25 min, 85–76% A for 25–27 min, 76–72% A for 24–45 min, 72-68% A for 45–60 min, 68–50% A for 60–75 min, and 50–97% A for 75–90 min. The flow rate was 0.7 mL/min with a sample injection volume of 10 μL. Temperature was maintained at 30 °C and detection wavelength was set at 296 nm.

#### 3.4.2. HPLC-MS Conditions

HPLC-MS analysis was conducted by AB Triple TOF 5600 plus Mass spectrometer (AB SCIEX, Framingham, USA) in positive electrospray ionization (ESI) mode with the liquid chromatography system (Waters Corp., Milford, MA, USA). Specific experimental methods and conditions were conducted as we reported previously [30]. The accurate mass and compounds’ structure was calculated by Peak View Software (AB SCIEX, version 1.2.0.3).

#### 3.4.3. Flow-Cytometric Analysis

Apoptosis degree was measured by Annexin V staining (Biosharp Biotechnology (Shanghai, China)). Firstly, A549 cells were incubated with the tested compound (arenobufagin) for 48 h, then collected from six-well plates, washed once with cold PBS (4C), and centrifuged (2000 rpm for 5 min) before being suspended with a 300 μL binding buffer. Annexin V-FITC (6 μL) was then added and the cells were stained in the dark for 15 min before adding 6 μL propidium iodide and 300 μL binding buffer. Apoptosis quantification was counted by flow cytometry (FC 500MCL, Beckman Coulter, Indianapolis, IN, USA).

#### 3.4.4. Western Blot Analysis

The cell samples were treated with arenobufagin (0, 5, 10, 25 ng) for 48 h. After incubation, the cells were suspended in lysis buffer (Tris-HCl, NaCl, EDTA, EGTA, NP-40 and PMSF) (Beyotime Institute of Biotechnology) on ice for 30 min and vortexed for 60 s, then centrifuged at 16,000 rpm at 4 °C for 20 min. Protein content was quantified by BCA assay kit (Thermo Fisher Scientific, Waltham, MA, USA). Protein samples with appropriate concentration were loaded on 10% sodium dodecyl sulfate-polyacrylamide gel electrophoresis (SDS-PAGE) gel in a 2 h run under 100 V. Subsequently, the proteins were transferred to polyvinylidene fluoride (PVDF) membranes by wet transfer electrophoresis. The membranes were blocked with skimmed milk before being incubated with the primary detection antibody PARP (Cell Signaling Technology, 1:1000), after being washed by TBS-T for 30 min. The membranes were then probed with counterpart secondary antibodies (Cell Signaling, 1:5000), and visualized by chemiluminescence (Bio-Rad, Hercules, CA, USA).

### 3.5. Cytotoxicity Assay

A-549 cell line were cultured in RPMI 1640 medium with 10% foetal bovine serum, 1% penicillin and streptomycin, and kept at 37 °C in an incubator with a 5% CO2 atmosphere. After being cultured for 24 h, the extracts were added and the cells were incubated for 72 h; 20 μL MTT (5 mg/mL) was then added and the cells were incubated for 4 h. 150 μL of DMSO was used to dissolve formazan crystals that were formed. Absorbance of the solution was measured with a spectrophotometer (Synergy 2, BioTek, Winooski, VT, USA) at 490 nm. The inhibition ratio was calculated by the following formula:

(2) $$\frac{\mathrm{OD}\;\mathrm{of}\;\mathrm{negative}\;\mathrm{control}-\;\mathrm{OD}\;\mathrm{of}\;\mathrm{experimental}\;\mathrm{group}}{\mathrm{OD}\;\mathrm{of}\;\mathrm{negative}\;\mathrm{control}-\mathrm{OD}\;\mathrm{of}\;\mathrm{blank}\;\mathrm{control}}\times 100\%$$

### 3.6. Spectrum-Effect Relationship Analysis

Based on the tested spectral and pharmacodynamic data, gray relational analysis, orthogonal partial least square (OPLS) regression, and pearson correlation analysis were applied to establish the spectrum-effect relationship and screen variables.

### 3.7. Statistical Analysis

Data were expressed as means ± standard error (SE). Statistical analysis was performed using Graph Pad computer software Version 7.00. The levels of significant difference were set at *p* < 0.05, *p* < 0.01.

## 4. Conclusions

In this study, the inhibitory effects of toad venom extracts on A549 cells were researched, and a spectrum-effect relationship analysis model was established with satisfactory fitting accuracy and forecasting precision, and utilized to screen the main bioactive components in toad venom extracts. The results showed that toad venom extracts with different proportions markedly inhibited the proliferation of non-small cell lung cancer cell (A549). Through further chemometrics and LC-MS analysis, a total of seven characteristic peaks were identified, of which arenobufagin (P7), telocinobufogenin (P13), and cinobufotalin (P16) were verified to have significant anti-cancer effects on several NSCLC cells. These constituents may have the potential to search for new compounds for cancer and other diseases. This study also revealed the putative mechanism that involves apoptosis via cleavage of PARP induced by arenobufagin in A549 cells. On account of the finding that some homologous recombination deficient tumors may depend on PARP-mediated DNA repair for survival, PARP inhibitors may increase tumor susceptibility to DNA-damaging agents. This study may provide a scientific foundation to further explore the mechanism of toad venom extracts in inhibiting the proliferation of cancer cells. Besides, it may also provide an eligible universal model for assessing the spectrum-effect relationship and screening of potential active agents in TCMs.

## Figures and Tables

**Table 1:** The areas of 19 common peaks from 21 batches of toad venom extracts samples.

**Peak No.**	**Retention Time (min)**	**Peak Area of Each Common Peak**										
		**Y1**	**Y2**	**Y3**	**Y4**	**Y5**	**Y6**	**Y7**	**Y8**	**Y9**	**Y10**	**Y11**
1	9.37	685.472	1265.932	1250.593	974.774	957.27	1010.413	867.402	1315.784	567.429	584.134	971.966
2	10.386	2341.575	1822.875	2126.622	2296.953	2570.147	1717.873	1900.818	2240.301	3425.94	862.656	2442.475
3	36.284	193.39	302.598	170.118	262.23	279.122	479.702	323.213	177.921	88.042	275.066	173.418
4	37.971	414.631	263.189	208.255	347.673	512.256	300.2	437.72	218.988	817.209	391.179	392.006
5	38.523	218.009	304.301	157.963	261.681	300.614	498.853	236.354	166.137	67.652	225.418	174.515
6	40.953	38.509	63.921	34.151	43.393	42.234	66.913	30.695	47.201	34.155	87.168	0
7	43.732	796.32	2388.288	644.745	1231.959	888.471	1855.31	1542.317	753.109	1129.193	4440.883	1147.35
8	44.934	336.203	705.949	289.553	264.788	264.326	554.939	327.15	330.268	324.53	776.276	206.247
9	46.02	71.965	158.015	123.149	113.564	74.126	157.364	123.155	133.014	73.051	224.505	73.266
10	46.371	178.196	53.935	123.241	123.454	190.555	117.823	84.01	114.243	70.535	91.672	168.053
11	52.959	108.922	127.318	110.642	90.331	76.243	92.967	114.004	120.607	83.299	192.508	100.575
12	56.132	278.008	103.346	58.983	137.138	162.316	181.121	122.917	44.306	69.387	196.867	168.316
13	59.667	500.784	638.751	462.998	393.104	661.84	667.263	425.983	484.238	466.795	791.954	390.518
14	63.809	705.163	603.384	757.856	620.893	536.75	631.009	793.538	804.449	665.587	722.991	726.219
15	66.06	74.869	137.608	86.244	67.821	42.847	118.539	112.336	95.842	55.426	194.199	76.422
16	67.864	869.363	862.359	956.45	818.259	962.528	833.387	721.204	1037.25	964.145	1261.244	805.822
17	74.282	970.366	671.157	892.667	814.774	802.975	708.759	947.063	943.754	784.519	915.178	973.719
18	78.438	1802.411	1060.794	1323.255	1400.663	1344.419	1077.235	1711.791	1420.933	1131.559	1510.696	1785.779
19	79.363	1114.966	245.608	1367.134	670.741	953.363	297.698	744.973	1482.98	1654.029	253.421	1032.58
**Peak No.**	**Retention Time (min)**	**Peak Area of Each Common Peak**										
		**Y12**	**Y13**	**Y14**	**Y15**	**Y16**	**Y17**	**Y18**	**Y19**	**Y20**	**Y21**	**CV(%)**
1	9.37	998.2	1125.908	1116.597	1222.232	885.401	947.145	601.786	796.456	1034.799	517.402	32.773
2	10.386	2203.685	1608.969	1919.392	1507.245	1448.348	2321.315	1767.301	3984.1777	2353.645	784.156	40.181
3	36.284	65.653	82.772	64.765	76.484	91.149	0	597.063	132.257	22.805	103.001	79.659
4	37.971	351.957	283.128	295.579	240.11	250.434	556.838	278.211	1056.185	518.699	315.577	53.743
5	38.523	15.176	89.527	22.878	78.507	84.599	27.898	836.463	127.611	20.978	32.686	101.301
6	40.953	34.931	64.09	62.812	61.77	67.033	0	60.759	30.906	25.364	81.67	48.868
7	43.732	1635.47	3586.32	3332.611	2859.296	3313.979	717.576	1623.332	1625.024	1175.547	4467.311	64.492
8	44.934	375.359	634.314	589.977	631.747	692.713	232.513	295.089	147.852	230.011	958.442	53.793
9	46.02	89.782	132.715	157.811	115.536	72.424	40.403	135.07	103.158	57.537	220.926	43.073
10	46.371	72.176	55.594	50.864	73.423	111.93	42.776	81.72	27.894	51.456	78.429	48.275
11	52.959	117.558	155.192	162.858	143.066	122.801	91.862	72.383	63.942	126.348	276.669	40.041
12	56.132	46.645	53.163	47.919	55.501	77.482	0	50.568	44.809	0	90.826	73.015
13	59.667	665.861	805.232	608.021	1151.574	1550.478	478.644	876.384	242.865	449.281	1101.234	49.667
14	63.809	789.828	481.103	601.599	487.236	426.751	976.433	687.474	621.274	849.146	1023.152	28.965
15	66.06	72.391	96.853	115.227	82.114	49.211	51.284	98.746	86.338	48.269	237.902	49.644
16	67.864	1366.021	1218.544	984.337	1268.317	1582.709	988.824	1173.91	621.845	1066.543	1880.648	33.838
17	74.282	894.732	534.174	647.725	531.19	447.47	1156.133	624.348	711.082	1222.557	1032.664	31.124
18	78.438	1628.258	785.547	922.434	665.363	439.385	2547.92	690.1	997.935	2615.188	1707.985	45.175
19	79.363	780.744	130.92	187.112	107.876	229.776	1226.677	148.282	2007.634	1501.246	329.411	75.945

CV(%) = standard deviation/the average value of the peak area × 100%.

**Table 2:** Similarity of fingerprints’ chromatogram of toad venom extracts from 21 batches.

Sample Number	Similarity	Sample Number	Similarity
S1	0.918	S11	0.945
S2	0.968	S12	0.984
S3	0.908	S13	0.906
S4	0.963	S14	0.934
S5	0.943	S15	0.918
S6	0.975	S16	0.878
S7	0.978	S17	0.892
S8	0.914	S18	0.928
S9	0.850	S19	0.771
S10	0.860	S20	0.921
		S21	0.865

**Table 3:** Score coefficient matrix of the chemical constituents.

Component					
Peak No.	1	2	3	4	5
1	−0.118	−0.152	−0.424	−0.527	−0.597
2	−0.866	−0.136	−0.192	0.359	0.095
3	0.168	−0.655	0.635	0.15	−0.235
4	−0.594	0.146	−0.086	0.698	0.306
5	0.101	−0.689	0.537	0.134	−0.224
6	0.908	−0.169	0.023	0.186	−0.004
7	0.896	0.193	−0.159	0.23	0.111
8	0.943	0.168	−0.072	0.017	0.066
9	0.781	0.09	0.341	0.289	−0.299
10	−0.121	−0.383	0.478	−0.548	0.453
11	0.759	0.601	0.061	−0.05	0.041
12	0.119	−0.332	0.662	−0.131	0.461
13	0.774	−0.095	−0.314	−0.192	0.313
14	−0.192	0.775	0.474	−0.019	−0.188
15	0.724	0.334	0.431	0.295	−0.167
16	0.702	0.386	−0.197	−0.165	0.28
17	−0.448	0.705	0.5	−0.181	−0.031
18	−0.479	0.689	0.395	−0.24	−0.021
19	−0.852	0.264	−0.001	0.181	0.092

**Table 4:** Total variance explanation of PCA.

Component	Initial Eigenvalues			Extraction Sums of Squared Loadings		
	Total	% of Variance	Cumulative %	Total	% of Variance	Cumulative %
1	7.696	40.507	40.507	7.696	40.507	40.507
2	3.599	18.942	59.449	3.599	18.942	59.449
3	2.686	14.137	73.586	2.686	14.137	73.586
4	1.699	8.943	82.528	1.699	8.943	82.528
5	1.339	7.047	89.575	1.339	7.047	89.575

**Table 5:** The inhibition rate of toad venom extracts ($\overset{-}{x}$ ± s, *n* = 3).

Sample No.	Inhibition Rate (%)
1	82.758 ± 1.192
2	89.852 ± 3.958
3	71.752 ± 4.590
4	82.715 ± 4.122
5	75.331 ± 11.816
6	87.305 ± 1.107
7	86.358 ± 5.267
8	75.634 ± 3.630
9	79.423 ± 9.368
10	96.727 ± 2.171
11	76.740 ± 7.320
12	84.24 ± 10.231
13	92.718 ± 4.874
14	91.383 ± 3.542
15	90.938 ± 7.262
16	90.677 ± 6.707
17	86.152 ± 4.317
18	84.362 ± 4.218
19	80.842 ± 6.811
20	88.343 ± 6.446
21	96.811 ± 0.330

**Table 6:** The gray relationship grade and their order between 19 peak areas and antitumor effect of toad venom extracts.

Peak Number	Gray Relation Grade	Order	Peak Number	Gray Relation Grade	Order
1	0.718	8	10	0.623	17
2	0.621	18	11	0.791	4
3	0.633	14	12	0.656	12
4	0.668	11	13	0.734	7
5	0.647	13	14	0.669	10
6	0.799	3	15	0.764	5
7	0.827	1	16	0.714	9
8	0.810	2	17	0.624	15
9	0.760	6	18	0.623	16
19	0.588	19			

**Table 7:** The correlation coefficients between characteristic peaks and A549 cell proliferation inhibition rate.

Peak No.	Pearson Correlation Coefficient	Peak No.	Pearson Correlation Coefficient
7	0.860 **	8	0.793 **
2	−0.677 **	6	0.637 **
16	0.548 *	9	0.524 *
12	−0.036	3	−0.102
17	−0.19	4	−0.222
11	0.696 **	19	−0.686 **
15	0.579 **	13	0.566 **
10	−0.494 *	14	−0.02
18	−0.106	5	−0.142
1	−0.238		

Notes: Pearson correlation, “r” represent the relevant strength; *, 0.5 ≤ ∣r∣ ≤ 0.8 indicates significant correlation; **, 0.8 ≤ ∣r∣ ≤ 1 indicates very significant correlation.

**Table 8:** MS data of 7 predicted active compounds (peaks) in toad venom.

Peak No.	t_R(min)_	MS^1^_(m/z)_	MS^2^_(m/z)_	Formula	Error (ppm)	Structural Identification
6	40.953	419.2405	401.2272; 371.2177; 365.2109; 353.2091; 335.1992	C_24_H_34_O_6_	−3.4	Hellebrigenol
7	43.732	417.2254	399.2151; 381.2046; 363.1945; 335.1998; 317.1893; 289.1944	C_24_H_32_O_6_	−3.0	Arenobufagin
8	44.934	417.2259	399.2154; 381.2054; 363.1949; 345.1844; 335.201; 317.1893	C_24_H_32_O_6_	−2.8	Hellebrigenin
11	52.959	473.2153	431.2061; 395.1847; 377.1739	C_26_H_32_O_8_	−3.4	19-oxo-cinobufotalin
13	59.667	403.2461	385.2359; 367.2256; 349.215; 321.2201; 303.2095	C_24_H_34_O_5_	−3.0	Telocinobufogenin
15	66.06	457.2233	415.2098; 397.1992; 379.189; 361.1786; 333.1840	C_26_H_32_O_7_	−3.7	19-oxo-cinobufagin
16	67.864	459.2353	417.2255; 381.2047; 363.194; 335.1995	C_26_H_34_O_7_	−4.6	Cinobufotalin

**Table 9:** The inhibitory effects of bufadienolides on A549, H157 cells.

IC_50_(ng/mL)	Arenobufagin	Telocinobufagin	Cinobufatolin
A549	12.530 ± 3.406	27.882 ± 17.291	23.082 ± 4.460
H157	8.908 ± 1.251	23.606 ± 7.381	131.123 ± 21.009

Data was presented as mean ± S.E. The experiments were performed at least three times. IC_50_ is expressed as the concentration of drug inhibiting cell growth by 50%.

## Supplementary Materials

Figure S1: the typical MS/MS spectrum of hellebrigenol; Figure S2: the typical MS/MS spectrum of arenobufagin; Figure S3: the typical MS/MS spectrum of hellebrigenin; Figure S4: the typical MS/MS spectrum of 19-oxo-cinobufotalin; Figure S5: the typical MS/MS spectrum of telocinobufogenin; Figure S6: the typical MS/MS spectrum of 19-oxo-cinobufagin; Figure S7: the typical MS/MS spectrum of Cinobufotalin; Figure S8: the supplementary information of WB.

## Abbreviations

BRCA	Breast cancer gene
CCA	Canonical correlation analysis
GC	Gas chromatography
GRA	Grey relational analysis
HPLC	High performance liquid chromatography
LC-MS	Liquid chromatograph-mass spectrometry
NSCLC	Non-small cell lung cancer
OPLS	Orthogonal partial least square
PARP	Poly (ADP-ribose) polymerase
PCA	Principal components analysis
RPAs	Relative peak areas
RRTs	Relative retention times
TCM	Traditional Chinese medicines
VIP	Variable importance in projection
