# Supplementary material for: Identification of Antitumor Constituents in Toad Venom by Spectrum-Effect Relationship Analysis and Investigation on Its Pharmacologic Mechanism
Source: Molecules. 2020 Sep 18;25(18):4269. doi: 10.3390/molecules25184269 (PMC7571126; doi:10.3390/molecules25184269)
Supplement: Supplementary file 1 [file molecules-25-04269-s001.pdf]

molecules

Supplementary Materials

Identification of Antitumor Constituents in Toad Venom by Spectrum-Effect Relationship Analysis and Investigation on its Pharmacologic Mechanism

Ji-Heng Wu, Yue-Ting Cao, Hong-Ye Pan and Long-Hu Wang *

College of Pharmaceutical Sciences, Zhejiang University, Hangzhou 310058, China; wujiheng@zju.edu.cn (J.-H.W.); caoyueting201314@zju.edu.cn (Y.-T.C.); 11819004@zju.edu.cn (H.-Y.P.)

* Correspondence: wang2000@zju.edu.cn; Tel.: +86-0571-88208455

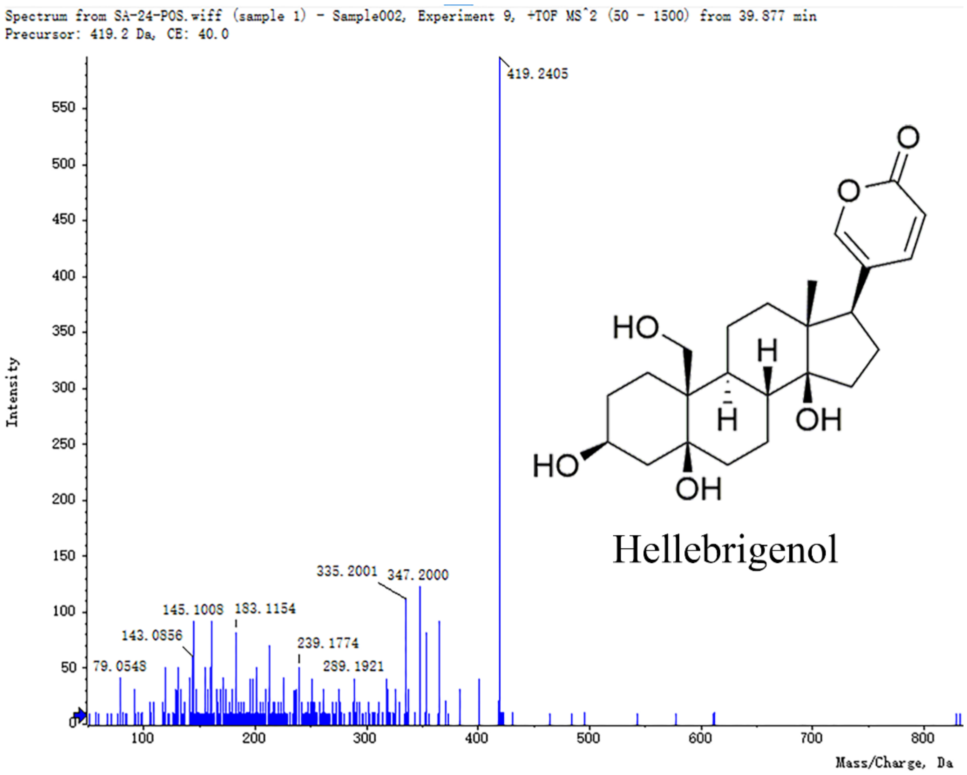

Figure S1: the typical MS/MS spectrum of hellebrigenol

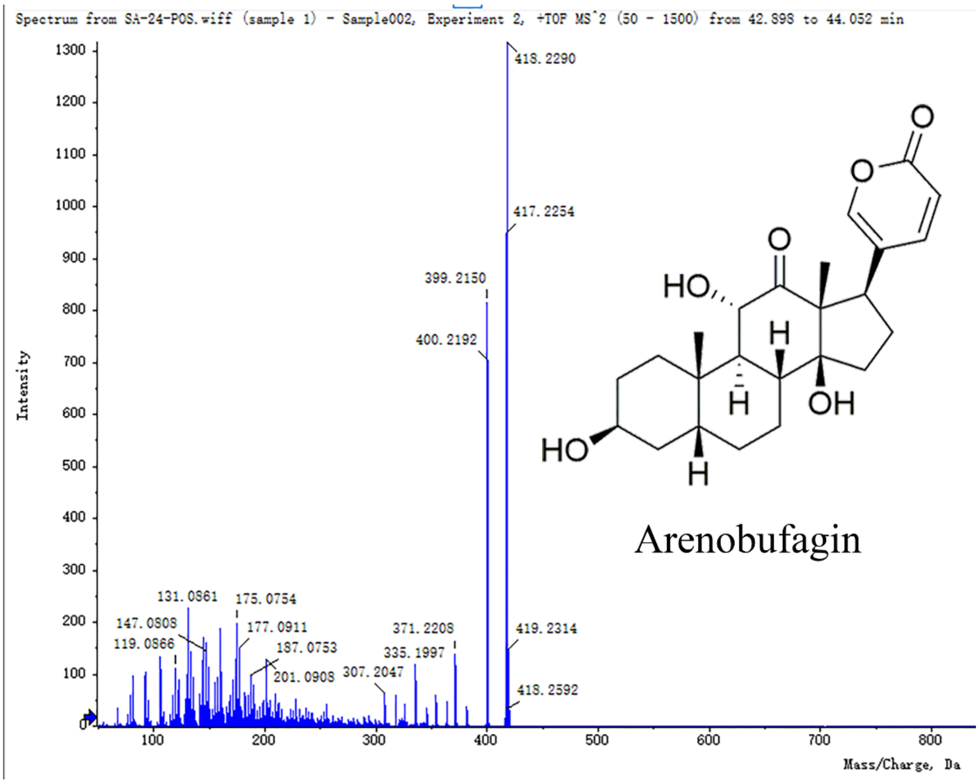

Figure S2: the typical MS/MS spectrum of arenobufagin

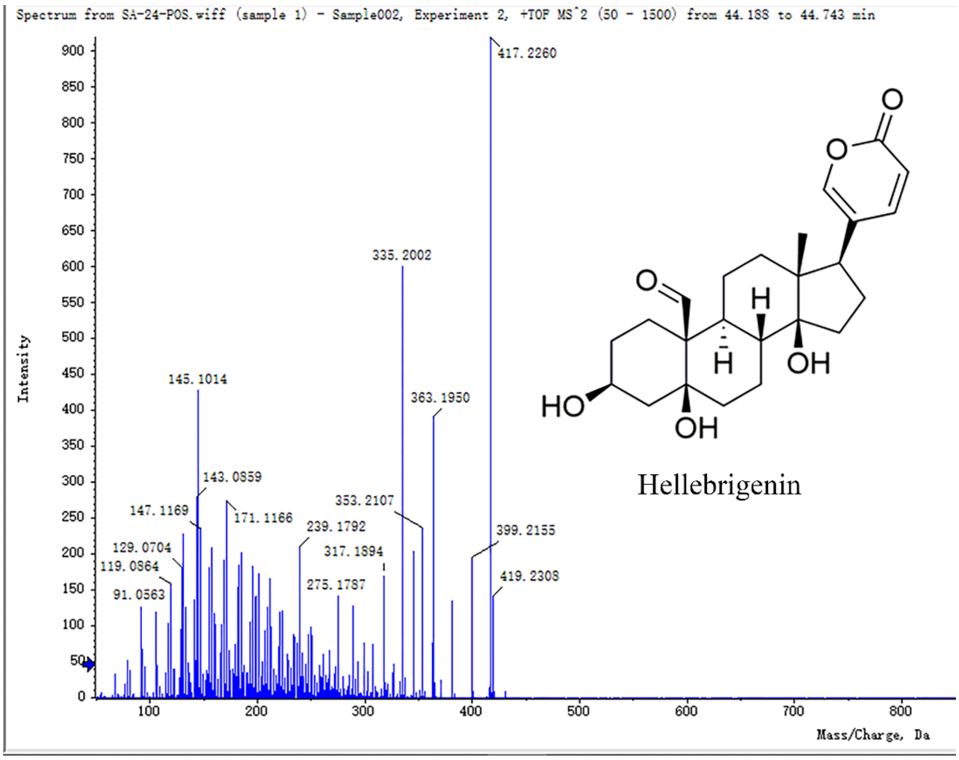

Figure S3: the typical MS/MS spectrum of hellebrigenin

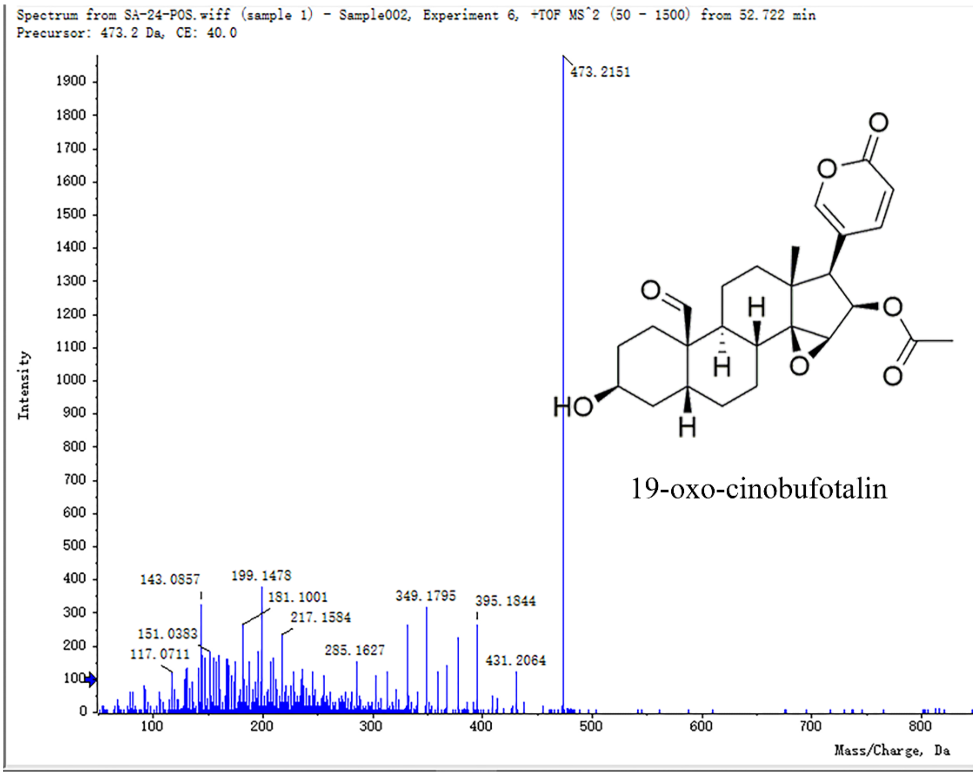

Figure S4: the typical MS/MS spectrum of 19-oxo-cinobufotalin

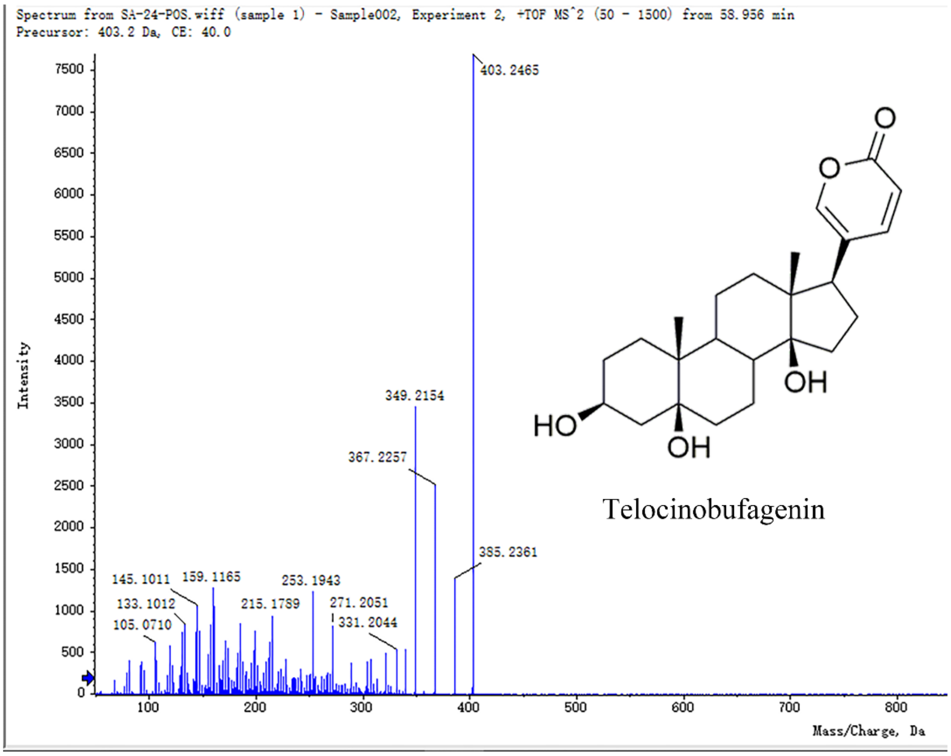

Figure S5: the typical MS/MS spectrum of telocinobufogenin

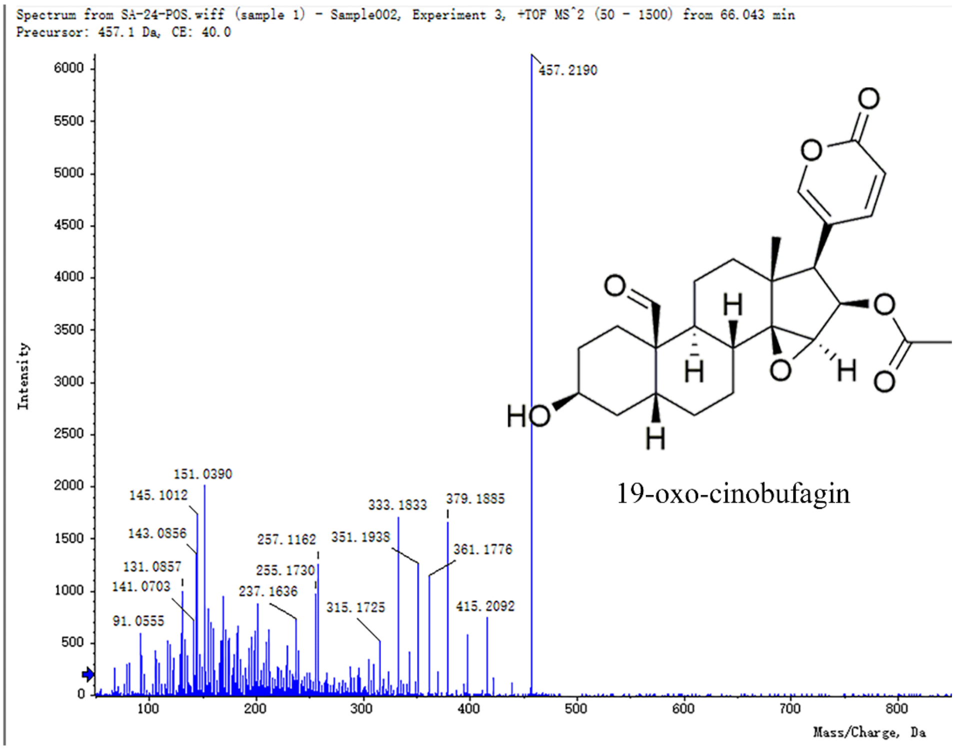

Figure S6: the typical MS/MS spectrum of 19-oxo-cinobufagin

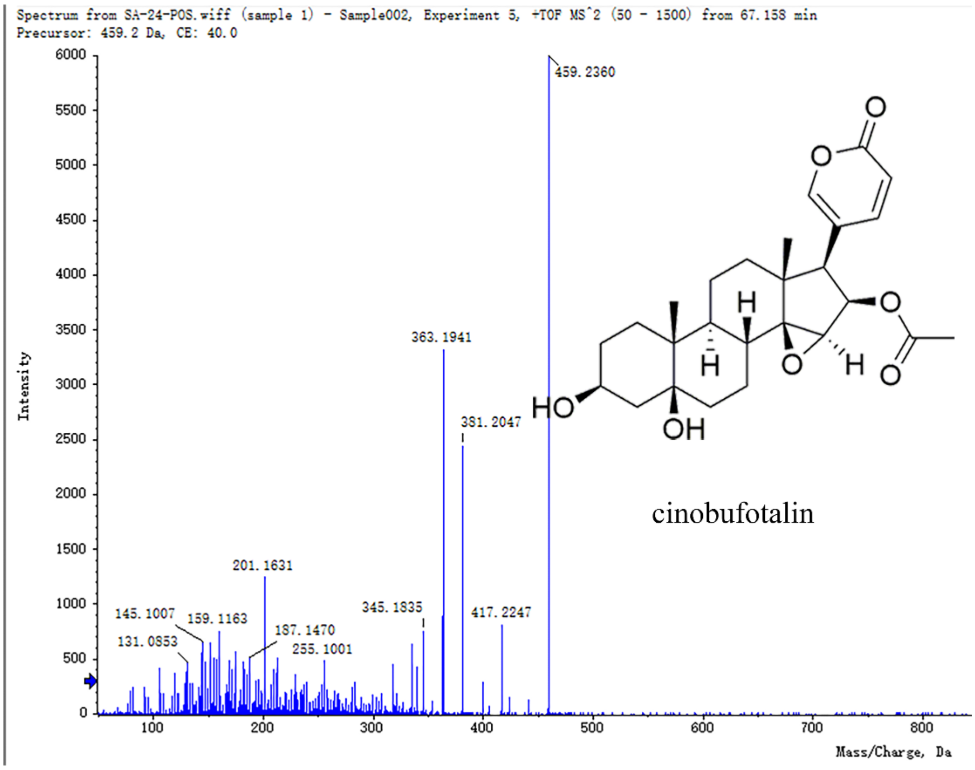

Figure S7: the typical MS/MS spectrum of Cinobufotalin

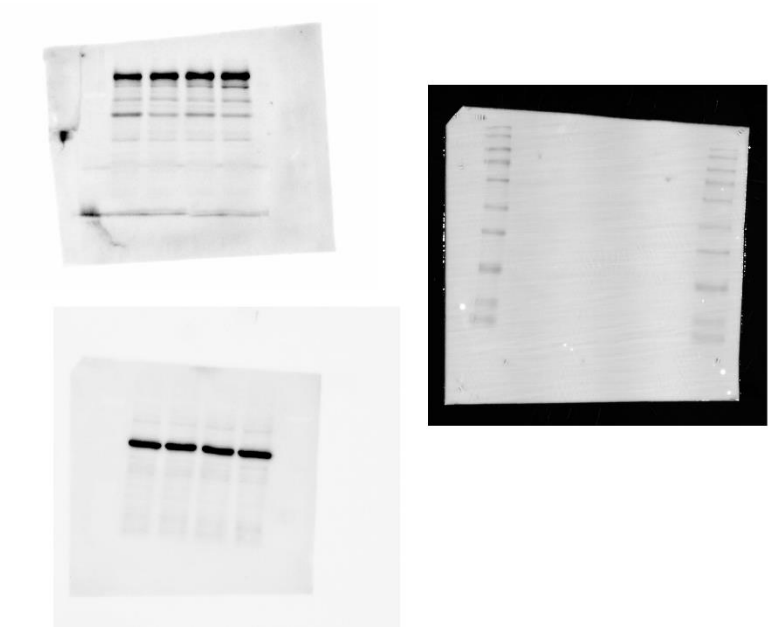

Figure S8: the supplementary information of WB
